# The immediate effect of facial candling on inflammatory mediators, substance P, symptoms severity, and quality of life in allergic rhinitis patients: Study protocol for a randomized controlled trial

**DOI:** 10.1097/MD.0000000000007511

**Published:** 2017-07-28

**Authors:** Nor Faizatul Fatikah Ismail, Chin Fen Neoh, Siong Meng Lim, Amir Heberd Abdullah, Mohd Fahmi Mastuki, Kalavathy Ramasamy, Nazli Zainuddin, Lokman Saim, Long Chiau Ming

**Affiliations:** a Faculty of Pharmacy, Universiti Teknologi MARA (UiTM), Puncak Alam; b Collaborative Drug Discovery Research (CDDR) Group, Pharmaceutical and Life Sciences CoRe, UiTM, Shah Alam, Selangor; c Environmetal Health Department, Faculty of Health Sciences, UiTM, Bertam, Penang; d Vector-Borne Diseases Research Group (VERDI), Pharmaceutical and Life Sciences CoRe, UiTM, Shah Alam, Selangor; e Medical Laboratory Department, Faculty of Health Sciences, UiTM, Puncak Alam, Johor; f Faculty of Medicine, UiTM, Puncak Alam, Selangor; g School of Pharmacy, KPJ Healthcare University College, Nilai, Negeri Sembilan, Malaysia.

**Keywords:** complementary therapies, ethnomedicine, folk medicine, immune system diseases, otorhinolaryngologic diseases, respiratory hypersensitivity, traditional medicine

## Abstract

**Introduction:**

Asian countries have a variety of ethnic groups and culture that provide their own traditional treatment in health care. Facial candling appears to be one of the popular traditional treatments in Southeast Asian. The complementary medicine practitioners promote that the facial candling treatment would help in reducing the symptoms of allergic rhinitis and other problems related to sinus. Due to the lack of evidence available, the effectiveness of this treatment method and its mechanism, however, remains unknown. The objective of this research is therefore to study impact of facial candling on inflammatory mediators, substance P (SP), symptoms severity, and quality of life (QoL) in allergic rhinitis patients.

**Method and analysis:**

A randomized, nonblinded, controlled trial will be carried out by recruiting a total of 66 eligible allergic rhinitis patients who fulfill the inclusion criteria from a university health center. The subjects will be randomly assigned into 2 groups: intervention group receiving facial candling treatment and control group (no treatment given). Samples of blood and nasal mucus will be collected right before and after intervention. Samples collected will be analyzed. The primary outcomes are the changes in the level of SP in both blood and mucus samples between both groups. The secondary outcomes include the levels of inflammatory mediators (ie, tumor necrosis factor alpha, interleukin (IL)-3, IL-5, IL-6, IL-10, and IL-13) and the severity of allergic rhinitis symptoms as measured by a visual analogous scale and QoL using the Rhinitis Quality of Life Questionnaire (RQLQ).

**Ethical and trial registration:**

The study protocols are approved from the Ethical and Research Committee of the Universiti Teknologi MARA (REC/113/15). The trial is registered under the Australia New Zealand Clinical Trial Registry (ACTRN12616000299404). The trial was registered on 03/07/2016 and the first patient was enrolled on 10/12/2016.

**Conclusion:**

Facial candling is one of the unique treatments using candles to reduce the severity of symptoms and inflammation. This is the first ever study conducted on facial candling that will give rise to new knowledge underlying the effects of facial candling on severity of symptoms and inflammation relief mechanism mediated by substance P and inflammatory mediators.

## 1 Introduction

In local Indonesian and Malaysian languages, allergic rhinitis is known as resdung. It is characterized by nasal symptoms that include posterior or interior rhinorrhea, nasal blocking, sneezing, and/or nose itchiness which present for more than 2 consecutive days with a minimum of 2 hours each day.^[1]^ Allergic rhinitis is a common disease globally including in the region of Asia.^[2]^ Several prevalence studies have shown that the annual prevalence ranged from 15.1% to 37.8% whereas the lifetime prevalence of allergic rhinitis ranged from 18.3% to 47.7% in the United Kingdom and Netherland population.^[3]^ Similarly, annual prevalence is 7.8% among adults in the United States (US) adults and 10% to 30% worldwide.^[4]^

Allergic rhinitis can cause serious implications to the mental health and physical status as it impairs quality of life (QoL), sleep, work, and school.^[5]^ Besides that, it significantly gives an impact on the expenses of health care. For instance the annual total direct and indirect costs of allergic rhinitis were approximately US dollar 2.7 billion and US dollar 1.4 billion in US and Sweden, respectively.^[6,7]^

Allergic rhinitis, one type of atopic syndromes, is a predisposition toward an exaggerated immunoglobulin E (IgE) mediated immune response in reaction to an environmental allergen such as food and allergen.^[8]^ Two main pathophysiologic mechanisms have been proposed. According to the first approach, in early phase of inflammation, the allergen activated B cell to produce IgE antibody.^[9]^ The IgE antibody then occupies the surface of mast cell which subsequently releases prostaglandin, histamine, and other chemical mediators into surrounding tissues. The release of mediators will then increase vascular permeability, vasodilatation, and increase mucus production,^[10]^ resulting in itching, sneezing, and congestion.^[11]^ Further release of inflammatory mediators promote a late-phase reaction which encourages the production of inflammatory mediators and hence, triggering the activation that causes the nasal mucosa cells to be assembled.^[12]^ The second approach suggests that allergic rhinitis is caused by an imbalanced T helper (Th)1 and Th2 cell-mediated response.^[13,14]^ Allergic rhinitis patients exhibit IgE, mast cell, and Th2 lymphocyte immune responses related to the early phase (symptoms: sneezing, nasal itching, runny, and congested nasal passages) as well as the late phase of the disease (symptoms: patient fatigue, malaise, and irritability).^[15]^ Th1 cells primarily secrete interleukin (IL)-2 and IL-3; whereas Th2 cells secrete IL-3, IL-4, IL-5, IL-10, and IL-13.^[16]^ Dominant Th2 cytokines can enhance allergen-specific IgE, which plays an important role in allergic inflammation.^[13,15]^

Previous study has shown that substance P (SP) involves in transference of noxious stimuli located in the spinal cord.^[17]^ SP is increasingly being recognized to play an important role in the inception of allergy and inflammation.^[18,19]^ In this case, SP increases immunoglobulin production, lymphocyte proliferation, and enhances cytokine secretion from monocytes, lymphocytes, macrophages, and mast cells. Apart from that, the release of inflammatory mediators including cytokines, histamine potentiates tissue injury, and stimulates further leukocyte recruitment, subsequently increase the inflammatory response.^[20]^

Most of the Asian countries are multiracial and cultural whom each community has specific method of traditional treatment.^[21]^ Facial candling, also known as *lilin resdung*, is one the traditional treatments used by the Indonesian and Malay ethnicities groups to cure or reduce various allergy and inflammation conditions including allergic rhinitis. In other Asian countries such as China and Japan, facial candling is practised in the form of moxibustion where burning cigar-shaped dried mugwort stick is used to warm regions and meridian points.^[22]^ Facial candling treatments are believed to give various impacts to the patients, including reducing stress as it promotes relaxation, and reduces headache, ear, or throat infection.^[23]^ Furthermore, the facial candling technique is said to improve the blood circulation throughout the whole body and reduce symptoms of allergic rhinitis. Usually after the facial candling treatment, there is white like powder left on the face of the clients which the practitioners insist that the white like powder is the evidence of fat eliminated during the process.^[24]^ However, there is a paucity of scientific evidence to support the execution of facial candling and the concept on how the treatment reduce and exert its effect on allergy and inflammation. There could be a link between the immediate effect of facial candling that could reduce the SP and inflammatory mediators in affected patients. It is also crucial to investigate the immediate effect of facial candling on symptoms severity and QoL on allergic rhinitis patients. Findings from this research will be instrumental to elucidate the value of facial candling on human pathological process.

## 2 Method and analysis

### 2.1 Study design and patient recruitment

A randomized, nonblinded, controlled trial will be carried out at a health clinic located at Universiti Teknologi MARA (UiTM), Puncak Alam, Malaysia. A total of 66 allergic rhinitis patients who have seen ear–nose–throat specialist at a university health clinic and fulfill the inclusion criteria will be invited to participate in this study. Skin prick and puncture test (SPT) was performed to confirm the diagnosis before enrolment confirmation by otolaryngologists to exclude patients with sinusitis and nasal polyps.

An otorhinolaryngology specialist will conduct confirmatory medical examination for the subjects and screening based on inclusion and exclusion criteria. The inclusion criteria are as follow: has a positive skin prick test, age between 18 and 65 years old, and negative urine pregnancy test for female. The participants will be excluded if they are diagnosed with chronic obstructive pulmonary disease, sinusitis/rhinosinusitis, nasal polyps, asthma, taking medication containing analgesic, antihistamine and steroid, and pregnant.

**Figure 1:**
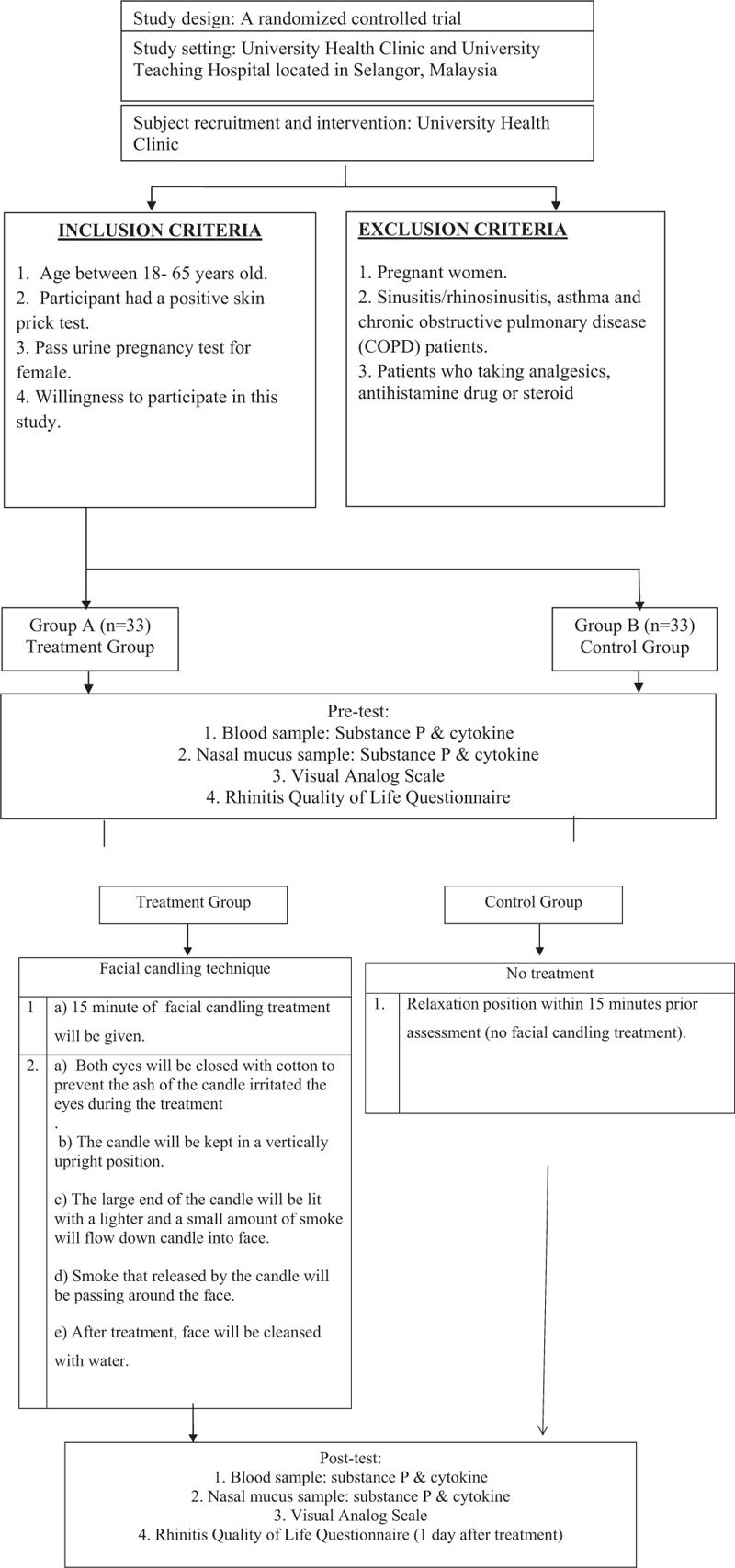
Study protocol.

An explanatory statement will be given and writing informed consent will be obtained prior to the commencement of the study. The participant will then be randomly assigned into 2 groups: allergic rhinitis patients or control groups (Fig. 1). Patients will be asked to choose among the sequentially numbered, sealed envelopes containing the group allocation, which will be determined by a computer-generated random number. An independent researcher produces the random numbers and prepares the envelopes. The current study is funded by UiTM and Ministry of Higher Education of Malaysia. Ethical approval has been obtained from the UiTM Research Ethics Committee (REC/113/115). The trial is registered under Australia New Zealand Clinical Trial Registry (ACTRN12616000299404).

### 2.2 Interventions

The facial candling treatment will be conducted for 15 minutes by a certified practitioner using a plain unscented facial candle. The facial candle is made of cloths which are soaked in beeswax. The facial candle is 9 to 10 inches in length and 0.25 to 0.5 inches in diameter. First, the patients will lie down on a massage table bed. Then, their both eyes will be closed with cotton to prevent the ash of the candle irritated the eyes during the treatment. Next, the candle will be kept in a vertically upright position and the large end of the candle is lit. After that, smoke that released by the candle will be passed around the patients face. Last, the face will be cleansed with water. Meanwhile, the control group will lie in a relaxation positions within 15 minutes prior assessment (no facial candling treatment) (Fig. 1).

### 2.3 Blood and mucus collection

Before and after the facial candling treatment, about 10 mL of venous blood will be taken by a certified phlebotomist. The whole blood will be collected into plain polyethylene tube until blood clot formation. After that, clots are separated from the wall of tube using a wooden applicator stick. Approximately 3 mL of nasal mucus will be collected with a suction from an aided vacuum that does not require chemical stimulation to prevent the introduction foreign substances into the nasal fluids. A small rubber-tipped vacuum device will be inserted gently inside the nasal passageways to stimulate nasal secretions. Both blood and nasal mucus samples will be collected and centrifuged (10,000 g and 4 °C for 15 minutes) to eliminate debris and cellular matter. The supernatant will be stored at −80 °C until analysis.

### 2.4 Outcome measures

The primary outcome measure for this study is the difference in the level of SP in blood and nasal mucus samples in pre- and postintervention. The secondary outcome measures include the levels of inflammatory mediators (tumor necrosis factor alpha [TNF-α], IL-3, IL-5, IL-6, IL-10, and IL-13) in both blood and nasal mucus samples. Other secondary outcome measures also include the visual analogous scale (VAS) score (indicating the pain intensity) and the Rhinitis Quality of Life Questionnaire (RQLQ).

### 2.5 Instruments

All samples will be analyzed individually using commercial human enzyme-linked immunosorbent assay kits (Table 1). The VAS is a measurement of pain and symptom intensity and has been found to be acceptable to patients and valid.^[25,26]^ The scale ranges is from “0” to “10” with a different facial expression whereby 0 represents “no pain/symptom” and 10 represents the “worst possible pain/symptom.” In this study, patients will be asked to rate their pain and symptom severity using the VAS pre- and postintervention. RQLQ will also be used to assess the disease-associated problems of the participants in 7 aspects of daily life: activities, sleep, nonnose/eye symptoms, practical problems, nasal symptoms, nose symptoms, and emotional symptoms. The score of each of the item is graded into 0 to 6. The total score of the 7 items will be calculated for every participant at baseline (1 day before) and 1 day after the intervention.

**Table 1:**
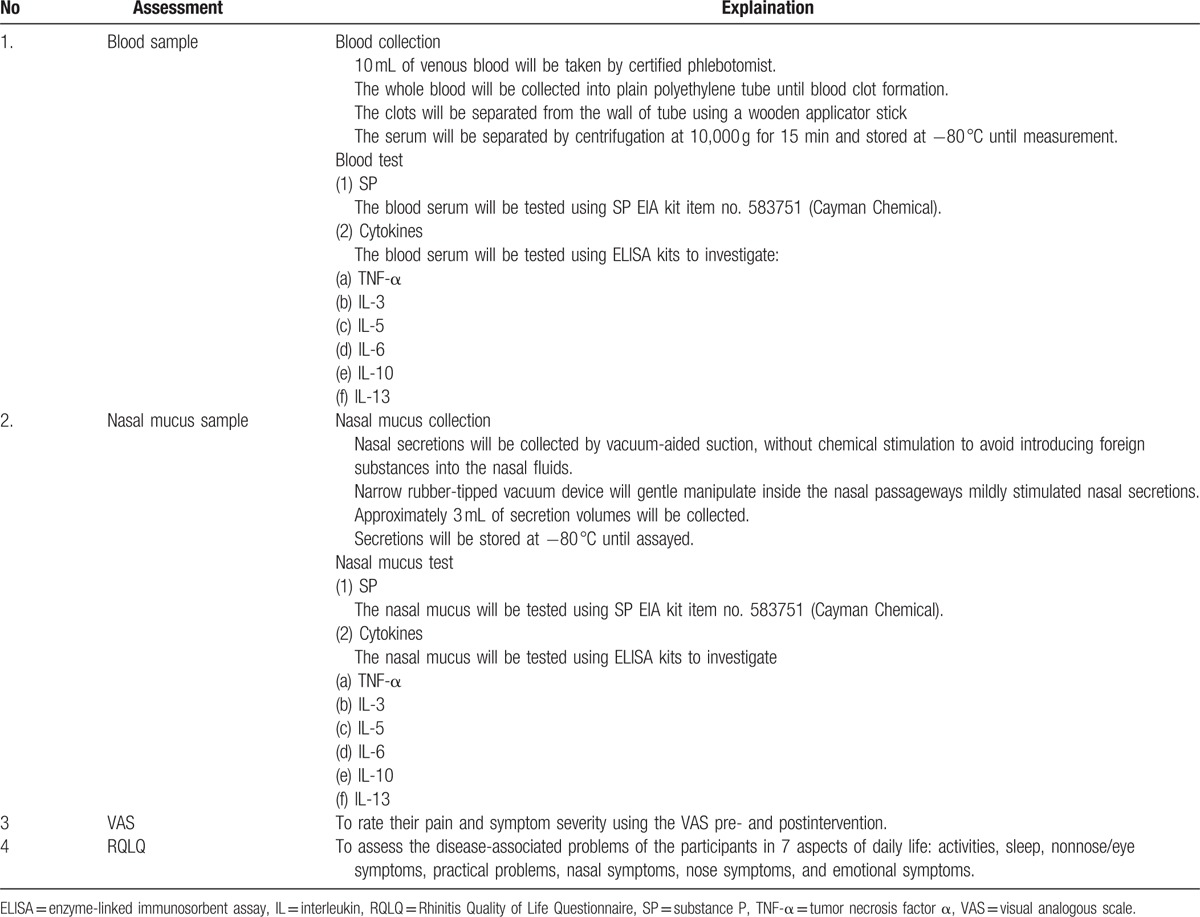
Analytical and assessment characteristics.

### 2.6 Sample size calculation

The sample size is calculated using Power and Sample Size software 3.1.2 version. The difference between the mean values of the intervention and control groups, and the standard deviation (postmassage) of SP levels^[27]^ will be used as parameters for sample size calculation given there is no previous finding of the immediate effect of facial candling treatment on SP. The mean levels of SP in the intervention group and control group are 50.43 and 56.27 pg/mL, respectively (the difference in the mean values between the experimental and control groups is 5.84 pg/mL).^[27]^ The type I error probability associated with this test of the null hypothesis is 0.05. A sample of 33 experimental subjects and 33 control subjects is therefore required to reject the null hypothesis that the population means of the experimental and control groups are equal, with a probability (power) of 0.8.

### 2.7 Data analysis plan

Data will be analyzed using SPSS software version 20.0. Mean, standard deviation, and range will be calculated for each variable. Data will be tested for normality; paired *t* test will be used (if the distribution is normal) or Mann–Whitney (if the distribution is skewed) to measure the differences pre- and postintervention within and between groups for every outcome measure. A *P* value of less than 0.05 will be used to show that the result is statistically significant.

### 2.8 Trial monitoring

The interim data and findings will be reported to the UiTM Research Ethics Committee. This is an investigator-initiated trial evaluating current. As there is minimal additional risk from a normal standard of care, a trial Data Safety and Monitoring Board was not deemed necessary.

## 3 Discussion

There is growing awareness that the knowledge of SP may help to develop new methods to treat pain and inflammation.^[19,28,29]^ For example, SP and inflammatory mediators appear to play a role in allergic rhinitis.^[30,31]^ Recently, the role of SP in the pathophysiology of clinical syndromes such as inflammatory rhinitis and diseases with chronic inflammatory pain in general is becoming clearer.^[32]^ It can be expected that the development of drugs and interventions aimed at the modulation of SP will be able to help in treatment of pain associated with these diseases. Previous study used the level of SP in nasal mucus to indicate the severity of pain in many chronic pain conditions, because the amount of SP is significantly greater in nose mucus than in plasma.^[33]^

As there is no previous finding related to facial candling, we explored studies that trialed effectiveness of other complimentary treatment methods. For example, 23 patients (19 were serologically tested positive for allergic diseases) with persistent allergic rhinitis were treated with 4 acupoint herbal plaster applied using the moxibustion technique. Clinical outcomes measured using the RQLQ indicated that the patients tested positively for allergic disease responded better to the treatment. Furthermore, IL-13 immune response was downregulated in that particular group of patients.^[22]^ Similarly, another study demonstrated the effectiveness of subcutaneous injectable immunotherapy in treating the sinus inflammation and pain in 281 allergic rhinitis patients. The subcutaneous immunotherapy as treatment of allergic rhinitis led to a reduction in all symptoms studied, improving the QoL of patients.^[34]^ Antiinflammatory effects of acupuncture and their relevance to allergic rhinitis have also been examined.^[35]^ Acupuncture has been shown to significantly reduce histamine-induced symptoms of allergic rhinitis. Furthermore, the study showed that there is significantly reduce of IL-4 and IL-10 in allergic rhinitis patients as acupuncture treatment has been applied^[36]^ and their finding was supported by Petti et al,^[37]^ who revealed the significant decrease of IL-10 immediately after acupuncture treatment. Interestingly, they showed unpredicted significant decrease of IL-2 and no significant changed in IL-6.

This is the first ever study conducted regarding facial candling treatment with the severity of symptoms (including nociception and pain) and inflammatory mediator. The present project will give rise to new knowledge underlying the immediate effect of facial candling on symptoms severity and inflammation relief mechanism caused by the reduction of SP and inflammatory mediators. Apart from that, this study will elucidate the extent to which facial candling can diminish the symptoms of allergic rhinitis thus improving patient's functions. Facial candling can be an alternative treatment approach and most likely popular intervention because it is a noninvasive technique which provides relaxation in face as well as reduction in allergy. Furthermore, the study helps the local traditional medicine practitioner generate new interests on treatment of other pain and inflammatory area such as osteoarthritis and migraine.

### 3.1 Strengths and limitations of this study

Facial candling can be an alternative treatment approach and most likely popular intervention because it is a noninvasive technique which provides relaxation in face as well as reduction in allergy.

The present project will be the first to provide new knowledge underlying the immediate effect of facial candling on symptoms severity and inflammation relief among patients with allergic rhinitis.

As the current scientific evidences are limited, this experimental study can provide necessary evidence to prescribe facial candling as a complementary and alternative treatment for allergic rhinitis.

## Acknowledgments

The authors thank Ministry of Higher Education and Universiti Teknologi MARA (UiTM), Malaysia for financial support for this research.
